# *TP53* gene mutation analysis in chronic lymphocytic leukemia by nanopore MinION sequencing

**DOI:** 10.1186/s13000-016-0550-y

**Published:** 2016-10-10

**Authors:** Crescenzio Francesco Minervini, Cosimo Cumbo, Paola Orsini, Claudia Brunetti, Luisa Anelli, Antonella Zagaria, Angela Minervini, Paola Casieri, Nicoletta Coccaro, Giuseppina Tota, Luciana Impera, Annamaria Giordano, Giorgina Specchia, Francesco Albano

**Affiliations:** Department of Emergency and Organ Transplantation (D.E.T.O.) Hematology Section, University of Bari, P.zza G. Cesare, 11 70124 Bari, Italy

**Keywords:** Chronic Lymphocytic Leukemia, *TP53*, MinION, Sequencing, Nanopore

## Abstract

**Background:**

The assessment of *TP53* mutational status is becoming a routine clinical practice for chronic lymphocytic leukemia patients (CLL). A broad spectrum of molecular techniques has been employed so far, including both direct Sanger sequencing and next generation sequencing. Oxford Nanopore Technologies recently released the MinION an USB-interfaced sequencer. In this paper we report our experience, with the MinION technology for the detection of the TP53 gene mutation in CLL patients.

Twelve CLL patients at diagnosis were included in this study. All except one patient showed the TP53 gene deletion in Fluorescence in situ hybridization experiments.

Patients were investigated for *TP53* mutation by Sanger and by MinION sequencing.

Analysis by Sanger was performed according with the IARC protocol.

Analysis by MinION was performed adopting a strategy based on long template PCR, read error correction, and post variant calling filtering.

**Results:**

Due to the high error rate of nanopore technology, sequence data were both used directly and before correction with two different *in silico* methods: ALEC and nanocorrect. A mean error rate of 15 % was detected before correction that was reduced to 4-5 % after correction.

Analysis by Sanger sequencing was able to detect four patients mutated for TP53. MinION analysis detected one more mutated patient previously not detected from Sanger.

**Conclusion:**

In our hands, the Nanopore technology shows correlation with Sanger sequencing but more sensitive, manageable and less expensive, and therefore has proven to be a useful tool for *TP53* gene mutation detection.

**Electronic supplementary material:**

The online version of this article (doi:10.1186/s13000-016-0550-y) contains supplementary material, which is available to authorized users.

## Background

Tumor-suppressor p53 gene (*TP53*) maps to chromosome band 17p13 and is pivotal for genome integrity. *TP53* encodes for the p53 protein, a transcription factor involved in essential cell functions, such as DNA repair, cell cycle control, apoptosis, aging, and stemness [1, 2]. Aberrant p53 function, due to 17p deletion (del(17p)) and/or *TP53* mutation, is associated with poor prognosis in chronic lymphocytic leukemia (CLL) patients [3–5]. Alterations of *TP53* occur in about 10 % of untreated CLL patients [6, 7], but up to 50 % in relapsed or refractory cases [8, 9]. Furthermore, over 80 % of cases harboring del(17p) also carry *TP53* mutations in the remaining allele [10, 11]. The frequency of mutations lacking del(17p) varies among different studies depending on the patient cohort and the methodology used, but in general it accounts for about 30 % of all *TP53* defects, while sole 17p deletions, without the *TP53* mutation, are less frequent, representing about 10 % of all *TP53* alterations [12]. Despite *TP53* mutation analysis is becoming a routine test for CLL patients, inconsistent results may be obtained among medical centers, possibly due to the variety of methods employed. To reduce the interlaboratory variability, in 2012 the European Research Initiative on CLL (ERIC) published recommendations (recently revised and available at http://www.ericll.org/pages/networks/*TP53*network) concerning several methodologies suitable for *TP53* analysis [13]. Two principal methodological procedures are suggested for *TP53* mutation detection: Sanger Sequencing or Next Generation Sequencing (NGS). Generally, at least exons from the fourth to the ninth, including splicing sites, should be covered in the analysis, even if the optimal range goes from the second to the eleventh. According to the ERIC recommendations on sensitivity threshold, only mutations detectable by Sanger sequencing and mutations with an allelic fraction higher than 10 %, if NGS methods are used, should be reported.

NGS is a powerful technology, allowing the detection of many low-rate mutations in potentially every type of disease and sample, but its major limitation remains the high initial investment required for the instrumentation setup. On the other hand, Sanger sequencing is a more affordable method but it is laborious, time consuming, and expensive over time. In this scenario, in 2012, Oxford Nanopore Technologies (ONT) released a portable sequencing device known as MinION [14] and in 2014 launched a community-focused access project: the MinION Access Programme (MAP). MinION is a single-molecule sequencer connected to a laptop through a USB 3.0 interface. Nanopore technology works by connecting two strands of DNA molecules by a hairpin, and sequencing them consecutively. During sequencing, the single strand of DNA passes through biologic nanopores on a chip, where an electric field is applied and electrical signal variations of consecutive 5-mers are recorded. DNA bases are then called using a cloud-based software (Metrichor). Template and complement sequences obtained are then used to generate the 2D high quality sequences. Typically, long reads are produced, up to some dozen kilobases. Due to the still high error rate, at the moment MinION performances cannot be comparable with the previous NGS platforms. However, the very low costs (estimated by the company around USD1000 when it will become commercially available), the ease of use, and the length of the reads, make MinION ideal for screening *TP53* mutations, followed by Sanger sequencing validation.

## Methods

### Patients

Twelve CLL patients at diagnosis were included in this study. All cases showed more than 70 % of lymphocytes in peripheral blood. All except one patient (CLL#7) showed the *TP53* gene deletion in Fluorescence in situ hybridization (FISH) experiments, performed as previously reported [15, 16]. The main clinical and biological features of these patients are reported in Table 1. All 12 samples were analyzed by Sanger and MinION in blinded manner.

**Table 1:** Patients clinical data and reads mapping statistics

Patient	Sex/Age	FISH	IgVH status	Stage (Binet)	Total 2D reads count	*TP53* mapped reads
CLL#1	F/62	del(17)(p13), del(13)(q14)	Unmutated	A	329	217
CLL#2	M/59	del(17)(p13), del(13)(q14)	Unmutated	B	307	173
CLL#3	M/59	del(17)(p13), del(13)(q14)	Unmutated	A	210	104
CLL#4	M/63	del(17)(p13), del(13)(q14)	Unmutated	A	288	194
CLL#5	F/65	del(17)(p13), del(13)(q14)	Mutated	A	212	188
CLL#6	M/72	del(17)(p13)	Mutated	A	342	173
CLL#7	F/30	del(13)(q14)	Unmutated	A	220	208
CLL#8	M/64	del(17)(p13), del(13)(q14)	Unmutated	B	154	135
CLL#9	M/56	del(17)(p13), del(13)(q14), del(11)(q22), +12	Unmutated	B	147	135
CLL#10	F/72	del(17)(p13), del(13)(q14)	Unmutated	B	197	90
CLL#11	F/45	del(17)(p13), del(13)(q14)	Unmutated	B	147	120
CLL#12	F/65	del(17)(p13), del(13)(q14)	Mutated	A	119	113

### Sanger sequencing

All samples were analyzed by direct Sanger sequencing according to the International Agency for Research on Cancer (IARC) protocol (http://p53.iarc.fr/Download/*TP53*_DirectSequencing_IARC.pdf). Electropherograms were then analyzed by visual inspection, glass free software for Sanger analysis data (http://shiny.bat.infspire.org/igcllglass/) and GeneScreen [17].

### *TP53* Amplification and barcoding

Genomic DNA was extracted from peripheral blood using the QIAamp DNA Blood Mini Kit (Qiagen) and quantified with Qubit 2.0 Fluorometer (Life Technologies).

According to the Oxford Nanopore Barcoding protocol for amplicons (Version DK003_1148_revB_12Feb2015), for each patient, we performed a Long-PCR using the PrimeSTAR GXL DNA Polymearse (Takara Bio Inc.), two tailed primers (PreBAR_P-559 5’-TTTCTGTTGGTGCTGATATTGCTCTCATGCTGGATCCCCACT-3’ and PreBAR_P-E11Re 5’-ACTTGCCTGTCGCTCTATCTTCTGACGCACACCTATTGCAAG-3’), 180 ng of genomic DNA, in a final volume of 25 μl. Thermal-cycling conditions were 98 °C for 10 s, 68 °C for 8 min (30 cycles) and 4 °C hold. The PCR products, visualized on an agarose-gel (1 %), were purified using the QIAquick PCR Purification Kit (Qiagen), and used as templates for the Barcoding PCR. Barcoding was performed with the same Polymerase and 12 different pairs of Barcoding primers from ONT, BC01-BC12, in a final volume of 50 μl. Thermal-cycling conditions were 98 °C for 10 s, 62 °C for 15 s, 68 °C for 8 min (15 cycles) and 4 °C hold.

### Minion library preparation and sequencing

Before starting library preparation, we purified, quantified and estimated the purity of samples (Nanodrop). Two mixes were then prepared, each pulling six barcoded amplicons at equal weight ratio. One microgram of each pool was diluted to 80 μl in nuclease-free water and prepared for sequencing. According to the ONT Sequencing protocol (SQK-MAP005), DNA was end-repaired with the NEBNext End Repair Module (New England Biolabs Inc.) and subsequently dA-tailed using the NEBNext dA-Tailing Module (New England Biolabs Inc.), prior to ligation of nanopore-specific adapters with Blunt/TA Ligase Master Mix (New England Biolabs Inc.). All purifications were performed with Agencourt AMPure XP beads (Beckman Coulter Inc.). Dynabeads His-Tag Isolation & Pulldown (Life Technologies) was used to elute the library in the pre-sequencing Mix. After the Platform QC run, sequencing mix (75 μl of 2X Running Buffer, 66 μl of nuclease-free water, 3 μl of Fuel Mix and 6 μl of the Pre-sequencing Mix) was loaded and the MAP_48Hr_Sequencing_Run.py protocol was started (MinION flowcell: FLO-MAP003; MinKNOW software: v48.2.14). During running, two reloads of the sequencing mix were performed and the run was stopped after 24 h.

### Data analysis

PoRe R package was used to obtain summary information about the sequencing data, as well as to extract FASTA files from FAST5 files [18].

The NanoOK package was employed for coverage and error assessment, using the *TP53* genomic sequence NC_000017.10 as reference [19].

Finally, Galaxy, a web-based platform for processing NGS data, was employed for variant analysis (https://usegalaxy.org/), [20–22]. Reads were aligned on GRCh37 human reference genome with the BWA-MEM method [23] using specific Nanopore platform parameters (https://github.com/lh3/bwa/blob/master/NEWS.md/#release-079-19-may-2014) and visualized with the Integrative Genomics Viewer (IGV) browser [24, 25]. Single nucleotide variants (SNV) and insertions/deletions (indels) detection was separately performed with the Varscan software [26], and VCF files were filtered and annotated with the gene variation IARC *TP53* database (v. R17). In particular, for SNV detection, the minimum read depth and supporting reads parameters were set according to the lowest mean coverage (minimum read depth of 50, minimum supporting reads of 10). Whereas, for indels detection, the minimum read depth and the minimum supporting reads values were set at 50 and 13, respectively. The minimum variant allele frequency threshold and *p*-value threshold for variant calling were 0.01 and 0.05, respectively, in all Varscan analyses.

## Results

### *TP53* mutation analysis by Sanger sequencing

All patients included in our study were investigated for *TP53* mutation by Sanger sequencing, according with the IARC protocol.

In the attempt of increasing our analytical sensitivity, we decided to test the MinION performance on selected CLL cases bearing the 17p deletion. As assessed by FISH, 11/12 patients carried the 17p deletion, and among them, 4 also had a *TP53* mutation (Tables 1 and 3). Reportedly [10], there is a high chance to find *TP53* mutations in patients with the 17p deletion, thus supporting our decision in case selection.

### *TP53* mutation analysis by MinION sequencing

*TP53* gene was amplified from genomic DNA in a single Long PCR reaction. Amplicons, spanning from exon 2 to exon 11 (7150 bp), were then barcoded and loaded on MinION for sequencing.

**Fig. 1:**
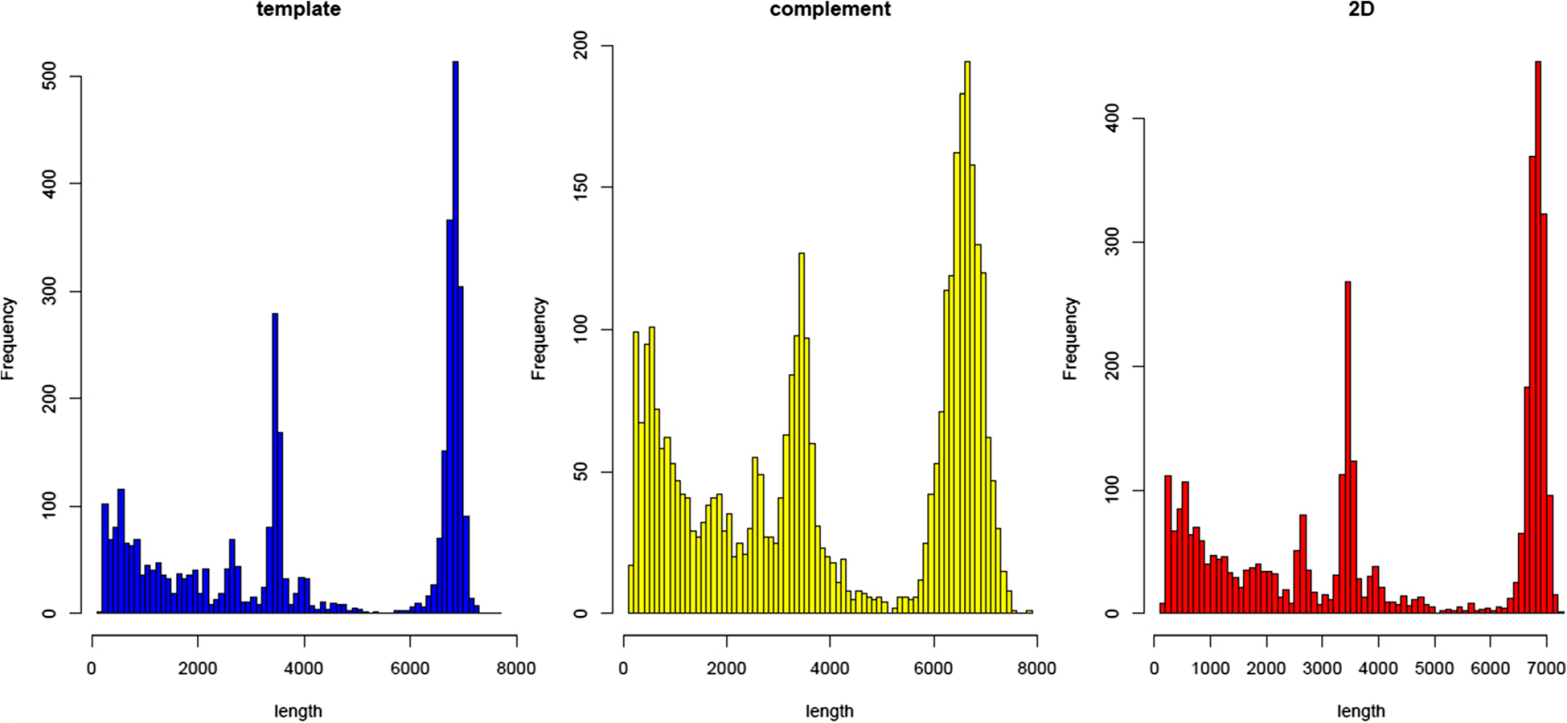
Reads length distribution plot

A total of two runs were performed, employing two libraries of six patients each one and two different flowcells, with 316 and 401 active pores respectively. For both experiments, 22421 fast5 total files were produced, containing raw electric signals. After base-calling in Metrichor, each run generated 14Mbases and 24Mbases. Finally, of the total reads produced, 3619 passed 2D filters and 2652 had a recognizable barcode (Table 2). In general, 2D pass reads had a length spanning from 150 bp to 7200 bp with a mean of around 4 Kb. Plotting the distribution of read lengths, we could observe a prevalent peak around 7 Kb, in agreement with our amplicon size (Fig. 1). Shorter reads, likely originating from breaks during purifications or library preparation, were observed as well.

**Table 2:** Reads summary from MinION sequencing

	Read type	Number of reads	Minimum length (bp)	Max length (bp)	Mean length (bp)
Pass	1D Template	3619	186	7607	4209
	1D complement	3619	173	7821	4047
	2D consensus	3619	176	7209	4170
Fail	1D Template	18319	5	199114	3407
	1D complement	7926	9	123134	3552
	2D consensus	4195	115	73841	3991

### Data analysis and error correction

Nanopore sequencing is a relative young technology and despite the rapid technical improvements, is still affected by a high error rate (estimated around 10 %), posing a problem in SNV analysis. To date, the only data on human gene variant analysis from MinION sequencing regard the detection of structural variants in cancer [27].

Among the several methods proposed to limit the error rate, we tested two *in silico* strategies: the Nanocorrect correction pipeline, specific for Nanopore data [28], and the more generic ALEC (Amplicon Long-read Error Correction) python script [29].

Nanocorrect is a pipeline inspired by pbdagcon (https://github.com/PacificBiosciences/pbdagcon) which uses DALIGNER (https://github.com/thegenemyers/DALIGNER) and poa [30] to correct sequencing errors in Nanopore reads. ALEC script has been originally developed for processing PacBio raw data to correct random sequencing errors specifically on long amplicons using alignment information from the SAM files [29].

After demultiplexing, reads were analyzed before and after error correction with NanoOK (Additional file 1: Table S1). A mean error rate of 15 % on mean values per 100 aligned bases counted including indels was detected before correction (raw reads), whereas the mean error rate dropped to 4-5 % after correction with nanocorrect and ALEC. Furthermore, separate error rate analysis for insertions, deletions and substitutions highlighted higher values for deletions. Mean insertion/deletion size reported was 1-2 nucleotides.

**Fig. 2:**
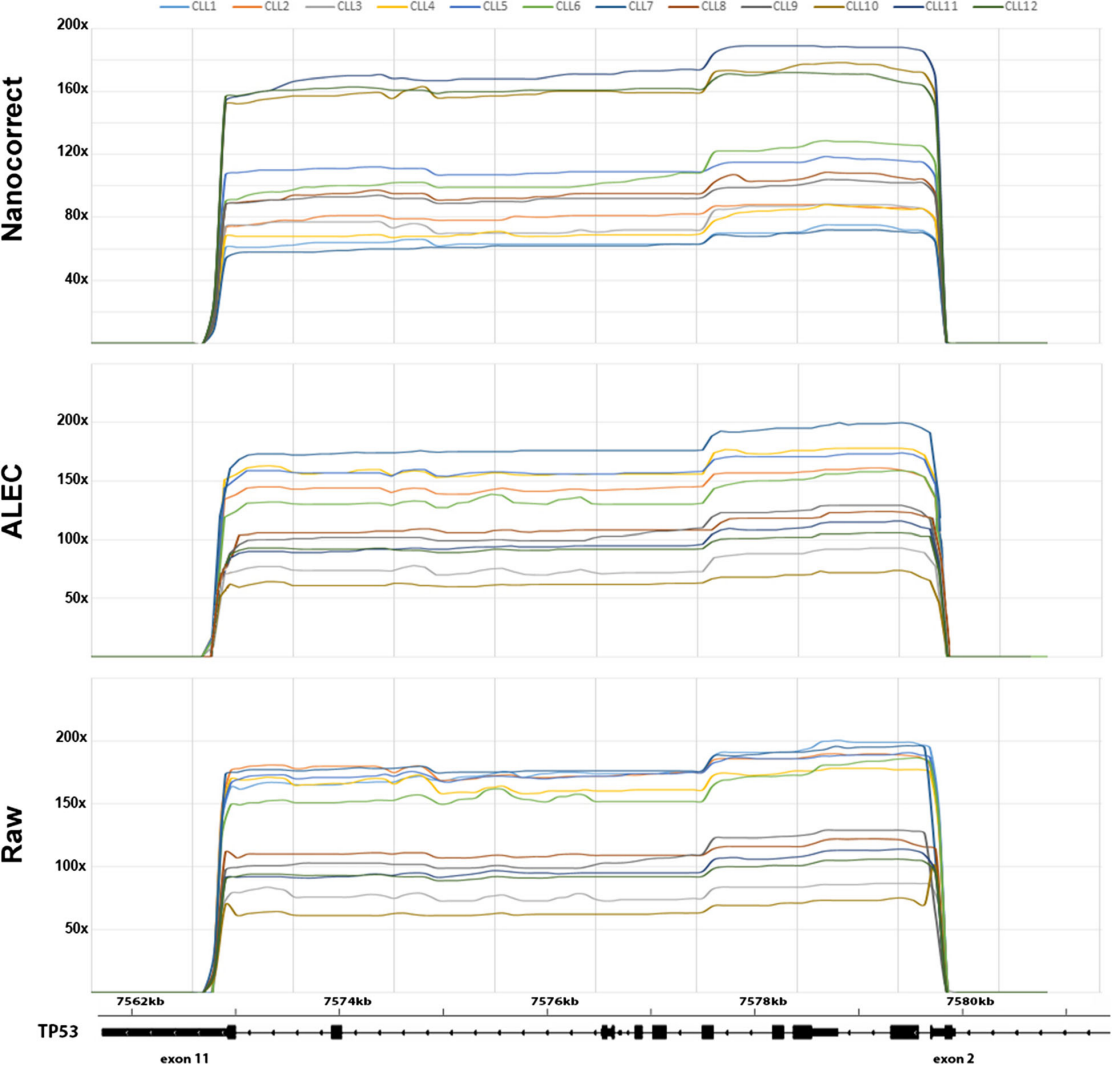
Coverage plots from corrected and uncorrected (Raw) reads after mapping on *TP53* genomic sequence

Coverage analyses showed that the *TP53* sequence, from exon 2 to exon 11, introns included, was completely and uniformly covered in each patient (Fig. 2). Moreover, as compared to Nanocorrect corrected reads, raw data and ALEC corrected data generally produced higher coverage rates, although the minimum coverage value was never below 50x.

### Variant calling and annotation

Read mapping, variant calling, annotation with “gene variation IARC TP53 database”, and variant filtering were performed with Galaxy starting from both raw reads and corrected reads. We created a complete workflow available *TP53* at https://usegalaxy.org/u/ematlab/w/tp53-mutation-screening-filtering to use with a reformatted file of “gene variation IARC TP53 database” (Additional file 2: File S2). After analysis the workflow outputs two files for SNV and Indels already filtered against *TP53* mutation IARC database.

### Variant filtering

Excluding intronic non-affecting splicing and exonic silent variations, analyses with raw reads returned the highest number of variants, especially for SNV. Conversely, the most stringent output was obtained from variant calling performed on nanocorrect corrected reads. Results were further filtered by Sanger sequencing detection limit, set around 15 % for SNV and 20 % for indels due to the higher error rate observed for deletions (Additional file 1: Table S1).

To evaluate whether recurrent sequencing errors could occur because of the nature of the sequences, thus producing false positives results, we checked if same variants were called in different patients. We found some recurrent variants both in samples sequenced on the same flowcell and on different flowcells, and therefore we decided also to filter variants by recurrence, considering the low chance to find the same mutation in more than one sample simultaneously.

Comparing the results obtained from corrected and uncorrected reads after filtering, we found 24 unique variants called from uncorrected reads, and 17 and 8 called from ALEC and nanocorrect corrected reads respectively.

A final summary of mutations detected with all methods we used and their comparison is shown in Table 3.

**Table 3:** Genomic and protein description of mutations detected by Sanger and MinION sequencing

Patient	Genomic description	Protein description	Exon/Intron	Functional Domain	Mutation frequency^a^	Raw Reads^b^	ALEC^c^	Nanocorrect^c^
CLL#2	g.7577144A > G	p.Leu265Pro	8-exon	DNA binding	34 %	38.6 %	39.13 %	78.72 %
CLL#8	g.7577548C > T	p.Gly245Ser	7-exon	DNA binding	31 %	26.9 %	30.28 %	ND
CLL#10	g.7577121_7577122GC > TT	p.Arg273Ser	8-exon	DNA binding	58 %	41.5 %	61.29 %	93.55 %
CLL#11	g.7577501del	p.Ser261Valfs^b^84	7-exon	DNA binding	46 %	39.7 %	44.21 %	35.48 %
CLL#12	g.7578406C > T	p.Arg175His	5-exon	DNA binding	ND	18.3 %	16.67 %	ND

*ND* not detected

^a^Percentage calculated from electropherograms using glass

^b^Frequency calculated starting from non-corrected reads

^c^Frequency calculated starting corrected reads

**Fig. 3:**
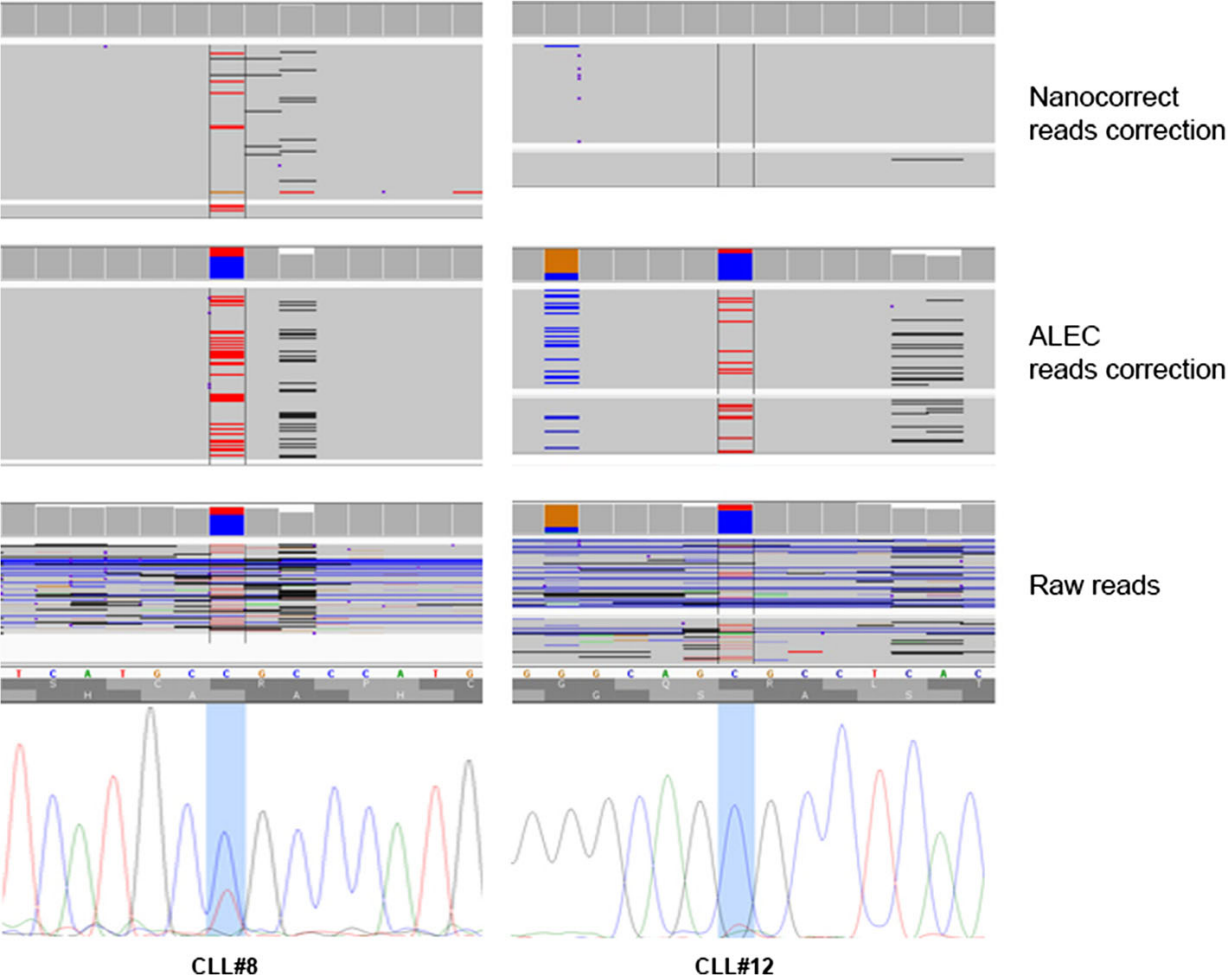
Example of mutations detected in two samples (CLL#8 and CLL#12). The mutation detected in CLL#8 was not found from nanocorrect corrected reads. CLL#12 mutation was detected only from the analysis with raw and ALEC corrected reads. The same mutation was barely visible in Sanger sequencing. Aligned reads are visualized by IGV

In general, all mutations detected by Sanger sequencing were also detected starting from raw reads and ALEC corrected reads. Additionally, for patient CLL#12, one mutation previously not detected by automated electropherogram analysis, was instead detected by visual inspection after the indications generated from MinION (Fig. 3, Table 3 and Additional file 3: Fig S3). Nanocorrect corrected reads produced two false negatives, namely in CLL#8 and CLL#12 (Table 3).

As expected, all recurrent variants resulted as false positive.

## Discussion

The MinION measures changes in electrical current as individual strands of DNA pass through one of its 500 protein tiny pores. To date, it is the only technology that directly measures a single DNA strand rather than incorporation events relative to a template strand. Moreover, speed, single-base sensitivity, and long read lengths make nanopores a promising technology for high-throughput sequencing [14, 31, 32]. In particular, the ability to generate long reads may help to resolve repeat regions within the genome that are challenging for short-read platforms; in fact, it has been recently demonstrated that MinION is able to determine the number of repeats in the human X chromosome repeat region using spanning reads of 42 Kbp [33]. This feature makes nanopore-based technology potentially very suitable for the targeted sequencing of cancer-relevant gene mutations. The only limitation of MinION technology is error proneness, an issue that can be moderated by several error correction bioinformatics methods.

In the present work we report the first use of MinION to detect *TP53* mutations in CLL patients. The recommended method to assess *TP53* mutation status is standard Sanger sequencing; pre-screening techniques such as denaturing high-performance liquid chromatography (dHPLC) or single-strand conformation analysis polymorphism (SSCP) could be performed to reduce time and costs. In any case results have to be confirmed by Sanger sequencing in order to identify the nature of mutation.

TP53 mutation analysis in CLL patients is particularly challenging considering that TP53 aberrations are infrequent at the time of initial chemotherapy, but increase their prevalence in multiply relapsed, chemotherapy-refractory populations [6, 8]. Therefore, monitoring TP53 status after the first line treatment or at CLL relapse is of considerable importance in order to plan the best treatment option for the patient.

**Fig. 4:**
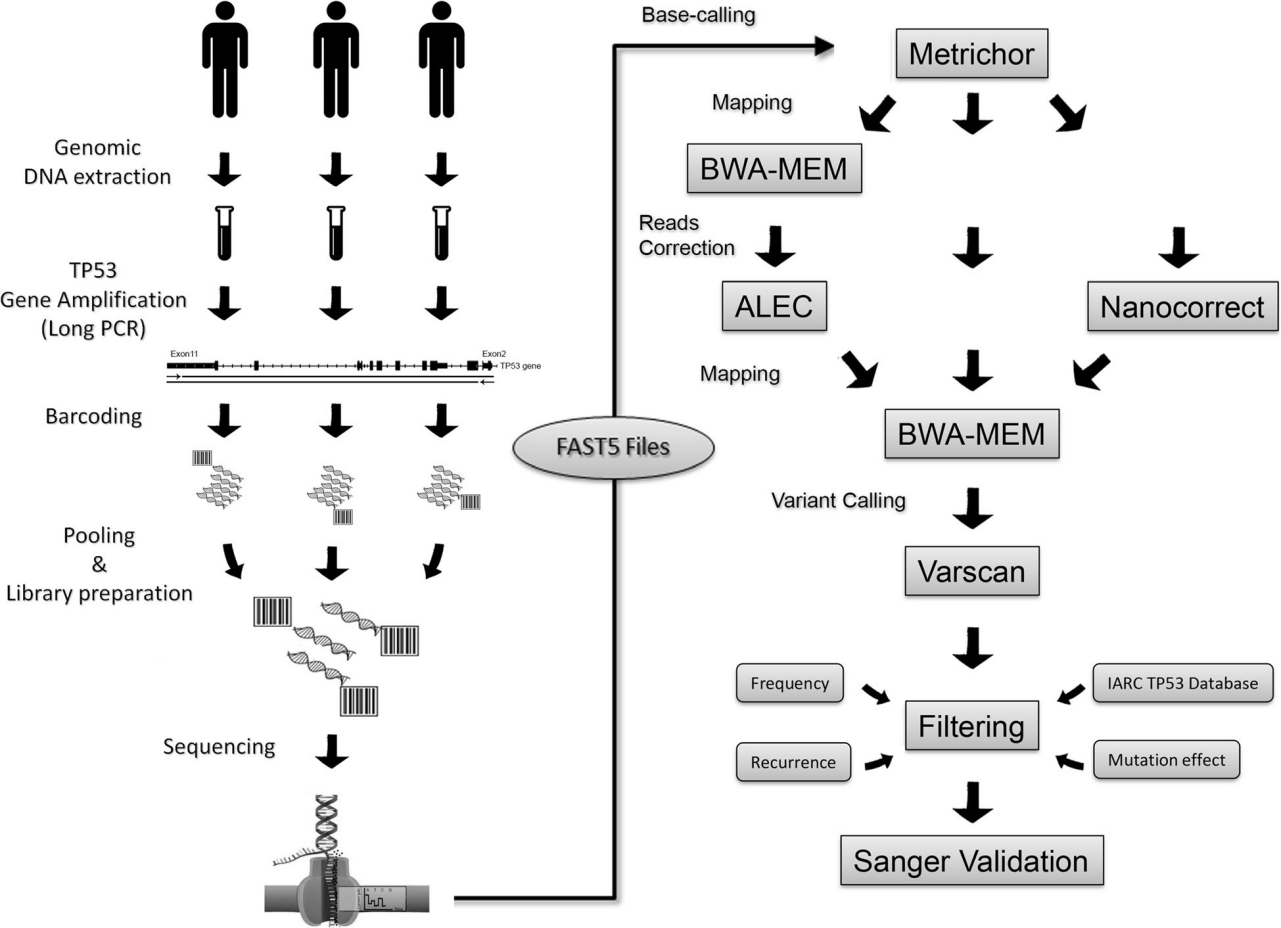
Schematic workflow implemented for *TP53* mutational analysis

Using our version of MinION (MIN-MAP001) we developed a workflow (Fig. 4) for read error correction together with a variant calling pipeline that considerably reduce the false positive variants, thus improving the overall performance. In our hands, the nanopore-based technology has proven to be a useful tool for *TP53* mutation detection. From a practical point of view, in the context of *TP53* mutational analysis, we suggest that MinION sequencing could be ideal in a pre-screening step, with several advantages compared to the current methods. Additionally, MinION pre-screening approach requires only two PCR per patient (*TP53* long amplification and barcoding) instead of one PCR per exon. The obtained results are more informative in comparison to those obtained with other pre-screening methods, potentially making the subsequent Sanger interpretation easier and more sensitive. Anyway at the state of the art of this technology, from a clinically point of view we recommend to comply at the ERIC reporting guidelines for Sanger sequencing (http://www.ericll.org/pages/networks/tp53network/ericmanualfortp53mutationalanalysis).

As mentioned, one of the major drawbacks is the high error rate that might lead to an elevated number of false positive variants. To limit this error, we tested two different *in silico* methods for reads correction: nanocorrect and ALEC, and applied additional filters on the variants called, such as IARC annotation, mutation effect, allelic frequency and recurrence. Mainly, recurrence was the most effective filter to reduce false positives rate, but required multiple samples to be performed.

As regards error correction methods, nanocorrect strongly reduced the final variants called, although produced some false negatives. Conversely, raw reads did not return false negative but too many false positives were called. The best results were obtained by ALEC, which returned no false negatives and a very limited number of false positives.

Finally, a relevant advantage of MinION is its limited costs. Excluding device charge, that is estimated around USD1000, we calculated that, if 6 samples are contemporarily run in a flowcell, the cost per sample is around USD180 a price comparable to that of Sanger sequencing. Moreover, since very high coverages were reached in our analyses, we believe that the overall costs could be further reduced by running more samples per flowcell.

## Conclusions

In summary, we demonstrate that MinION is a suitable tool for the detection of *TP53* gene mutations in patients affected by CLL, and propose that its low costs and ease of use may potentially expand its field of applications to other cancer genes. At the moment MinION is still not adequate for substituting Sanger sequencing nor other NGS technologies, but can be a useful strategy for pre-screening analyses. Finally, the constant improvements of nanopore technology promise an exclusive and convenient use of MinION in the immediate future.

## Additional files

Additional file 1: Table S1. Error analysis results on raw and corrected reads. (XLSX 11 kb)
Additional file 2: Reformatted file of “gene variation IARC *TP53* database” to use in Galaxy workflow for variants annotation. (TXT 1603 kb)
Additional file 3: Figure S3. Sanger analysis on CLL#12 showing the mutation g.7578406C > T. It is not detectable by the analysis software due to the low intensity of variant peak. The presence of the peaks for T base both in forward and in reverse sequences confirm the mutation. (TIF 3259 kb)

## Abbreviations

ALEC: Amplicon long-read error correction
BC: Barcode
bp: Base pair
BWA-MEM: Burrows-wheeler aligner – maximal exact match
CLL: Chronic lymphocytic leukemia
del(17p): 17p deletion
dHPLC: Denaturing high-performance liquid chromatography
DNA: Deoxyribonucleic acid
dNTP: deoxynucleotide triphosphate
ERIC: European Research Initiative on CLL
FISH: Fluorescence in situ hybridization
IARC: International Agency for Research on Cancer
IGV: Integrative Genomics Viewer
Indel: Insertion/deletion
Kb: Kilobase pair
MAP: MinION access program
NGS: Next-generation sequencing
ONT: Oxford nanopore technologies
PCR: Polymerase chain reaction
SNV: Single nucleotide variant
SSCP: Single-strand conformation analysis polymorphism
TP53: Tumor protein p53
VCF: Variant call format
