# Supplementary figures and images for: TP53 gene mutation analysis in chronic lymphocytic leukemia by nanopore MinION sequencing
Source: Diagn Pathol. 2016 Oct 10;11:96. doi: 10.1186/s13000-016-0550-y (PMC5057401; doi:10.1186/s13000-016-0550-y)

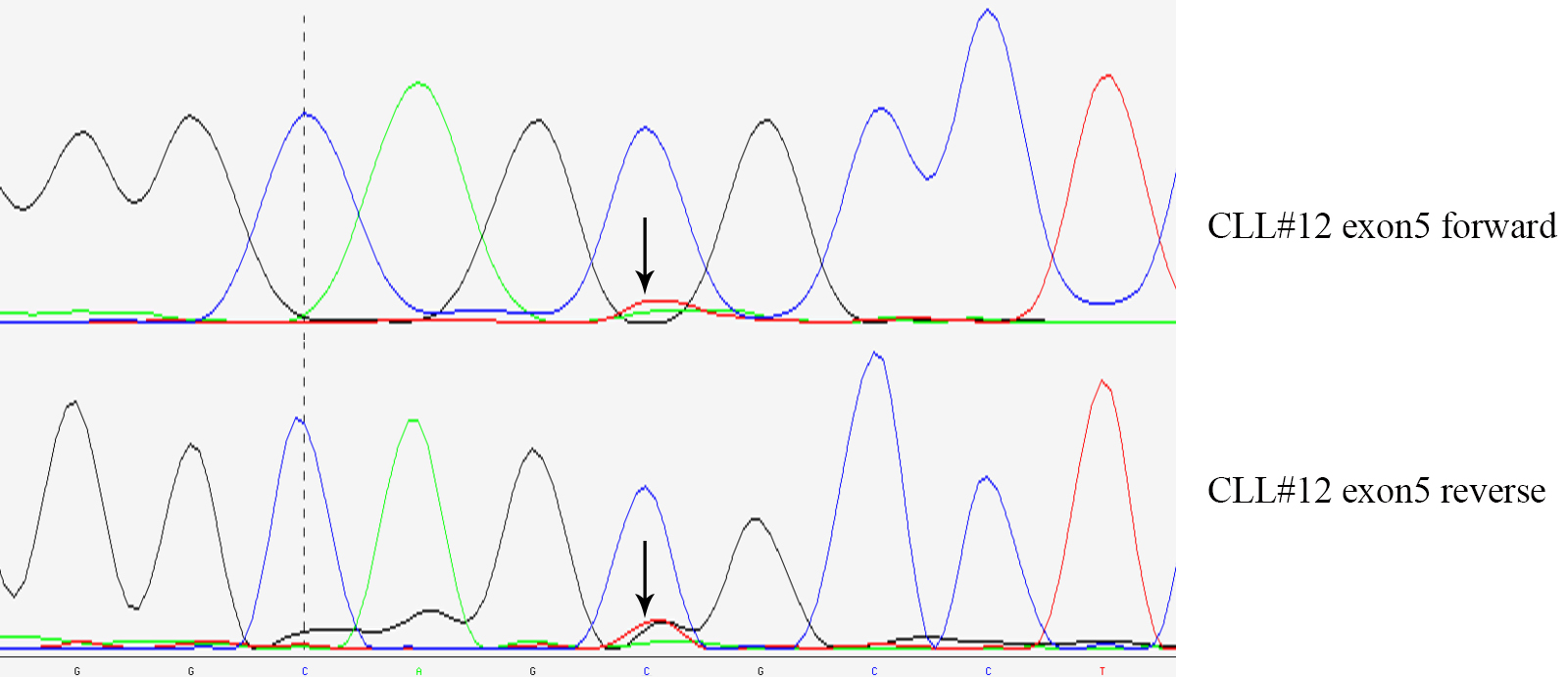

Supplement: Additional file 3: Figure S3. — Sanger analysis on CLL#12 showing the mutation g.7578406C > T. It is not detectable by the analysis software due to the low intensity of variant peak. The presence of the peaks for T base both in forward and in reverse sequences confirm the mutation. (TIF 3259 kb) [file 13000_2016_550_MOESM3_ESM.tif]
